# Adaptación de los factores de mal pronóstico en pacientes con COVID-19 al escenario actual

**DOI:** 10.23938/ASSN.1029

**Published:** 2023-03-09

**Authors:** María José Núñez Orantos, Francisco Javier Candel, Juan González del Castillo

**Affiliations:** 1 Servicio de Medicina Interna Hospital Clínico San Carlos Madrid España; 2 Servicio de Microbiología Clínica Hospital Clínico San Carlos Madrid España; 3 Servicio de Urgencias Hospital Clínico San Carlos Madrid España; 4 Instituto de Investigación Sanitaria del Hospital San Carlos Madrid España

Sr. Editor:

Hemos leído con interés el trabajo de Blanco-Taboada y col^1^, publicado recientemente en su revista, que describe diferentes factores de riesgo que actúan como predictores independientes de mortalidad o ingreso en la Unidad de Cuidados Intensivos (UCI) en pacientes hospitalizados por COVID-19. La posibilidad de estratificar el riesgo de mala evolución de estos pacientes de acuerdo a estos factores nos parece fundamental para evitar altas inadecuadas e ingresos innecesarios, por lo que consideramos que este tipo de trabajos resultan de gran interés. Desde fases muy tempranas de la pandemia de COVID-19 se publicaron diferentes artículos que identificaban factores de riesgo de mala evolución de los pacientes^2^^,^^3^ y modelos predictivos de mortalidad^4^^-^^6^ para tratar de ayudar en la toma inicial de decisiones. No obstante, a la hora de decidir si aplicarlos en la práctica clínica o no, deben valorarse algunas cuestiones.

En primer lugar, hay que valorar las variables de resultado que se tienen en cuenta. La mortalidad a 30 días es una variable que clásicamente se ha utilizado en los estudios de factores de riesgo para valorar el efecto de una intervención sobre el paciente. Sin embargo, durante la pandemia hemos asistido a estancias muy prolongadas de los pacientes, por lo que el desenlace mortal podría ocurrir transcurridos más de 30 días desde la atención inicial. Por otra parte, la supervivencia final del paciente no implica que no haya sufrido un cuadro grave que haya requerido su ingreso en la UCI o la instauración de tratamientos intensivos como ventilación mecánica u oxigenoterapia de alto flujo. Por lo tanto, a la hora de valorar los factores de riesgo en esta enfermedad, con estancia prolongada en pacientes graves, deberían evaluarse otras variables de resultado distintas a la mortalidad. De hecho, si los pacientes no se recuperaron ni murieron durante el periodo de estudio, sus datos no deben excluirse del análisis, o se debería considerar un periodo apropiado para el análisis del evento. En este sentido, nos parece de gran interés la descripción de factores de riesgo de ingreso en UCI que realizan Blanco-Taboada y col^1^.

En segundo lugar, hay que valorar el método de inclusión de pacientes. En es el estudio de Blanco-Taboada y col^1^ se han incluido únicamente pacientes con una decisión ya tomada de hospitalización. Esto significa que los factores de riesgo pueden no ser útiles para la toma inicial de decisiones en entornos como urgencias o atención primaria. Lo ideal, en nuestra opinión, hubiese sido incluir todos los pacientes evaluados en el centro hospitalario independientemente de la decisión inicial de ingresarlos o no. No obstante, la mayoría de los factores de riesgo obtenidos coinciden con los de las otras series publicadas, como son la edad, la saturación de oxígeno, o los niveles de creatinina o de proteína C reactiva, lo cual indica que los resultados podrían generalizarse a otros niveles asistenciales.

En tercer lugar, quizá el aspecto más importante en el cambiante escenario que es la pandemia de COVID-19 es la identificación del contexto en que se ha realizado el estudio. El periodo de inclusión de pacientes del estudio de Blanco-Taboada y col^1^ comprende desde el 1 de marzo de 2020 hasta el 9 de febrero de 2021. En el verano de 2022 existen dos aspectos fundamentales que condicionan el pronóstico de los pacientes: por un lado, la variante circulante (omicron) ha mostrado un comportamiento menos virulento, habiéndose descrito menores tasas de hospitalización, de ingreso en UCI, de necesidad de ventilación mecánica y de mortalidad^7^; por otro lado, la elevada tasa de población vacunada ha afectado de forma importante el pronóstico de los pacientes, haciendo que el riesgo de complicaciones disminuya drásticamente^8^. Por tanto, extrapolar los resultados de un estudio realizado antes de que se dieran las condiciones del momento actual puede no ser adecuado ya que las circunstancias del virus y del huésped son distintas, lo que puede influir en los resultados. Por este motivo, de cara a realizar una toma de decisiones con seguridad es necesario validar en el escenario actual los factores de riesgo y/o escalas pronósticas obtenidas en otros contextos.

Son escasos los artículos científicos publicados que evalúan los factores de riesgo en este nuevo escenario; tan solo dos de ellos^9^^,^^10^ los analizan en el contexto de elevadas tasas de vacunación. Ambos estudios establecen que la edad, el estado inmunológico y la comorbilidad (enfermedad cardiaca, hepática, renal y pulmonar crónica) continúan siendo los factores de riesgo predictores de mala evolución, aunque este riesgo es mucho mayor en población no vacunada. No obstante, estos dos estudios se realizaron cuando la variante circulante era delta y, por tanto, aún carecemos de información del efecto de la variante omicron sobre los factores de riesgo.

Antes de trasladar las conclusiones de los estudios de escalas pronósticas y/o de factores de riesgo a la práctica clínica habitual se deben tener en cuenta diferentes aspectos que determinen la seguridad de esa decisión (Tabla 1).

**Tabla 1:** Aspectos a tener en cuenta para valorar si los resultados ofrecidos por cualquier estudio son adecuados para nuestra población y, por tanto, trasladables a nuestra rutina asistencial

Aspectos	Observaciones
Variable de resultado utilizada	Esta puede ser mortalidad intrahospitalaria, mortalidad a 30 días, necesidad de oxígeno de alto flujo, necesidad de ventilación mecánica, ingreso en la unidad de medicina intensiva.
Identificación del contexto	Atención Primaria, urgencias, hospitalización, ingreso en la unidad de medicina intensiva.
Escenario global	Tasa de vacunación, variable circulante, disponibilidad de tratamientos comercializados o no
Método de inclusión de los pacientes	Idealmente deben ser consecutivos
Características de los pacientes incluidos	Debe incluir información clara del paciente en el momento de ser incluido en el estudio: comorbilidad, punto del curso de su enfermedad, o la situación de gravedad.
Intervenciones realizadas que pudieran modificar los resultados	¿Están todos los pacientes bajo el mismo protocolo de tratamiento?
Utilidad de las predicciones	¿Satisfacen la necesidad clínica?
Tamaño muestral y número de eventos	El tamaño de la muestra y el número de eventos observados (resultado de interés) deben ser suficientes para asegurar la potencia y relevancia de los resultados.
Comportamiento de las variables	Los análisis de regresión logística implican la categorización de las variables continuas cuando el incremento del riesgo no es lineal; el desafío consiste en determinar cuántas categorías deben establecerse para cada variable y cuáles son sus puntos de corte. Esta categorización puede provocar una pérdida en la precisión del modelo obtenido.
Validación de los resultados	Idealmente debe realizarse una validación externa (con datos distintos a los empleados para la obtención del modelo y/o de los predictores).

En conclusión, creemos que son necesarios nuevos estudios que validen en la situación actual de la pandemia COVID-19 los factores de riesgo y las escalas pronósticas previamente publicadas.
