# Supplementary material for: Adjustment of poor prognostic factors in patients with COVID-19 to the current scenario
Source: An Sist Sanit Navar. 2023 Mar 9;46(1):e1029. [Article in Spanish] doi: 10.23938/ASSN.1029 (PMC10123388; doi:10.23938/ASSN.1029)
Supplement: Related texts

# Reply: Influencia de distintos escenarios sobre los factores de mal pronóstico en pacientes con COVID-19

**DOI:** 10.23938/ASSN.1033

**Authors:** Ana Laura Blanco-Taboada, María del Rocío Fernández-Ojeda, María Milagros Castillo-Matus, María Dolores Galán-Azcona, Javier Salinas-Gutiérrez, María Victoria Ruiz-Romero

**Affiliations:** 1 Servicio de Medicina Interna Hospital Universitario Virgen de la Victoria Málaga España; 2 Unidad de Medicina Interna Servicio de Medicina Hospital San Juan de Dios de Aljarafe Bormujos Sevilla España; 3 Unidad de Calidad e Investigación Hospital San Juan de Dios de Aljarafe Bormujos Sevilla España

Sr. Editor:

Hemos leído con gran interés la carta de Núñez Orantos y col^1^, *Adaptación a los factores de mal pronóstico en pacientes con COVID-19*^2^. En ella, los autores comentan diferentes aspectos a valorar a la hora de extrapolar escalas pronósticas y factores de riesgo a la práctica clínica habitual en una población y contexto determinados.

La mortalidad en pacientes con COVID-19 ventilados mecánicamente con insuficiencia respiratoria aguda grave ha sido muy alta, siendo un factor muy importante a considerar^3^. Coincidimos con Núñez Orantos y col en que estos pacientes han requerido en ocasiones un ingreso hospitalario prolongado, lo que afecta a la relevancia de la variable de estudio de mortalidad a los 30 días. Por ello, es importante que nuestro estudio^2^ describa los factores de riesgo de ingreso en la Unidad de Cuidados Intensivos (UCI), ya que la asistencia con ventilación mecánica u oxigenoterapia de alto flujo ha sido clave en el tratamiento de pacientes con COVID-19. Por tanto, el análisis de los factores de riesgo de ingreso en UCI, las complicaciones durante la hospitalización, factores analíticos, días de ingreso en UCI, utilización de ventilación mecánica no invasiva, invasiva u oxigenoterapia de alto flujo, son variables clave a tener en cuenta, distintas de la mortalidad, que pueden ser muy útiles en nuestra práctica clínica diaria.

En cuanto al método de inclusión de pacientes, en nuestro estudio solo incluimos los pacientes que se encontraban hospitalizados en la planta de Medicina Interna y que habían sido ingresados desde el área de Urgencias, no los pacientes atendidos en Atención Primaria o en Urgencias que fueron dados de alta a domicilio. Coincidimos con Núñez Orantos y col^1^ en que incluir a todos estos pacientes para poder identificar a aquellos con mal pronóstico a fin de poder realizar una derivación precoz es de gran relevancia.

Nos parece muy importante la última reflexión realizada, ya que nos encontramos ante un cambio continuo de variantes del virus SARS-CoV-2 que condicionan, por tanto, la pauta de tratamiento, las complicaciones y el pronóstico de los pacientes. Durante las primeras olas de la pandemia, antes de la introducción de las vacunas, era esencial poder identificar a las personas con COVID-19 con mayor riesgo de complicaciones, por lo que se han publicado abundantes estudios al respecto^4^^-^^7^, e incluso se han desarrollado herramientas de evaluación de riesgos^8^. Pero en la fase temprana de la cuarta ola se ha observado un patrón de características en pacientes hospitalizados con COVID-19 diferente al de olas anteriores, con resultado de pacientes más jóvenes con menos comorbilidades, menos hospitalizaciones, menos diagnósticos respiratorios y una disminución en la gravedad y la mortalidad^9^.

A este cambio de patrón se suma una alta tasa de población vacunada, lo que conlleva menores tasas de ingreso hospitalario, mejor pronóstico final de estos pacientes y menor mortalidad. Los efectos positivos de la campaña de vacunación se están visualizando en muchas partes del mundo, pero la desaparición de esta infección aún está lejos de ser una realidad, ya que también se ve amenazada por la presencia de nuevas variantes del SARS-CoV-2 que podrían socavar la eficacia de la vacuna y, por tanto, encontrarnos ante un continuo cambio de escenario con necesidad de nuevos estudios^10^.

Diferentes estudios han identificado factores de riesgo clínicos en pacientes vacunados; la edad^11^, la obesidad, las condiciones médicas preexistentes y la desventaja socioeconómica^12^^,^^13^^)^ parecen afectar a la respuesta a algunas vacunas o medicamentos inmunosupresores^13^^,^^14^. Se han descrito algunos algoritmos, como el QCovid3 en Reino Unido^15^, que discriminan los pacientes vacunados con mayor riesgo de ingreso hospitalario y mortalidad. Esta información puede ayudar a planificar el reclutamiento de los pacientes, a priorizar el refuerzo vacunal y a administrar futuros tratamientos específicos, realizando una clasificación de los pacientes según sus características que podríamos tener en cuenta también para realizar nuevos estudios.

Como sabemos, con la propagación global del SARS-CoV-2 las variantes virales surgieron rápidamente, prevaleciendo sobre las cepas originales encontradas al comienzo de la pandemia^16^, entre ellas la de nuestro estudio (realizado desde el 1 de marzo de 2020 hasta el 9 de febrero de 2021). Hay diferentes mecanismos involucrados en la aparición de nuevas mutaciones, como la selección natural, las infecciones persistentes en pacientes inmunocomprometidos y los cambios de huésped. El cambio continuo de variedades hace que sea difícil extrapolar los resultados de nuestro estudio^2^ a la situación clínica actual. Sin embargo, nuestro estudio fue de gran utilidad en estas primeras olas de la pandemia -de gran incertidumbre sobre las pautas de nuevos tratamientos y la necesidad de ingreso en la UCI- para disminuir las complicaciones, la mortalidad y conseguir una mejora en la calidad asistencial. En el contexto de una pandemia grave causada por un virus nuevo, es vital abordar las lagunas de conocimiento e identificar factores potencialmente predictivos de complicaciones de la COVID-19, lo que justifica una mayor investigación^17^.

Actualmente, viviendo en un continuo cambio de variantes, creemos también que es muy necesaria la realización de nuevos estudios que permitan la validación de los factores de riesgo en cada escenario para obtener escalas pronósticas que se adecúen a la virulencia del SARS-CoV-2, al estado inmunológico poblacional y a las manifestaciones clínicas más frecuentes, para dar a la población el mejor tratamiento posible y mejorar con ello la asistencia hospitalaria.

Tenemos que tener en cuenta que la publicación de modelos predictivos para la COVID-19 está aumentado rápidamente^18^ para respaldar la toma de decisiones médicas en un momento en que se necesitan con urgencia, como ocurrió en los primeros momentos de la pandemia. Pero estos modelos tienen a su vez un alto riesgo de sesgo debido a los puntos anteriormente comentados, por lo que no se pueden tomar como objeto de actuación o guía de decisiones clínicas. Los predictores identificados en los modelos deben considerarse, por tanto, predictores candidatos para nuevos modelos actuales, siguiendo una metodología adecuada y teniendo en cuenta todas aquellas características descritas por Núñez Orantos y col^1^.

Queremos dar las gracias a Núñez Orantos y coautores por la gran reflexión realizada, y animar a los centros hospitalarios a realizar este tipo de estudios, cada vez más completos, para dar respuesta a las nuevas incertidumbres que esta pandemia nos presenta.

## References

[1] Núñez Orantos MJ, Candel FJ, González del Castillo J (2023). Adaptación de los factores de mal pronóstico en pacientes con COVID-19 al escenario actual. An Sist Sanit Navar.

[2] Blanco-Taboada AL, Fernández-Ojeda MR, Castillo-Matus MM, Galán-Azcona MD, Salinas-Gutiérrez J, Ruiz-Romero MV (2022). Factores de mal pronóstico en pacientes hospitalizados por COVID-19. An Sist Sanit Navar.

[3] Ibarra-Estrada MÁ, García-Salas Y, Mireles-Cabodevila E, López-Pulgarín JA, Chávez-Peña Q, García-Salcido R (2022). Use of airway pressure release ventilation in patients with acute respiratory failure due to COVID-19: results of a single-center randomized controlled trial. Crit Care Med.

[4] Berenguer J, Ryan P, Rodríguez-Baño J, Carcas A, Jarrín I, Yllescas M (2020). Characteristics and predictors of death among 4035 consecutively hospitalized patients with COVID-19 in Spain. Clin Microbiol Infect.

[5] Pan F, Yang L, Li Y, Liang B, Li L, Ye T (2020). Factors associated with death outcome in patients with severe coronavirus disease-19 (COVID-19): a case-control study. Int J Med Sci.

[6] Wu C, Chen X, Cai Y, Xia J, Zhou X, Xu S (2020). Risk factors associated with acute respiratory distress syndrome and death in patients with coronavirus disease 2019 pneumonia in Wuhan, China. JAMA Intern Med.

[7] Yang X, Yu Y, Xu J, Shu H, Xia J, Liu H (2020). Clinical course and outcomes of critically ill patients with SARS-CoV-2 pneumonia in Wuhan, China: a single-centered, retrospective, observational study. Lancet Respir Med.

[8] Clift AK, Coupland CAC, Keogh RH, Diaz-Ordaz K, Williamson E, Harrison EM (2020). Living risk prediction algorithm (QCOVID) for risk of hospital admission and mortality from coronavirus 19 in adults: national derivation and validation cohort study. BMJ.

[9] Maslo C, Friedland R, Toubkin M, Laubscher A, Akaloo T, Kama B (2022). Characteristics and outcomes of hospitalized patients in South Africa during the COVID-19 omicron wave compared with previous waves. JAMA.

[10] Rotondo JC, Martini F, Maritati M, Mazziotta C, Di Mauro G, Lanzillotti C (2021). SARS-CoV-2 infection: new molecular, phylogenetic, and pathogenetic insights. Efficacy of current vaccines and the potential risk of variants. Viruses.

[11] Wall EC, Wu M, Harvey R, Kelly G, Warchal S, Sawyer C (2021). Neutralising antibody activity against SARS-CoV-2 VOCs B 1.617.2 and B.1.351 by BNT162b2 vaccination. Lancet.

[12] Hoes J, Boef AGC, Knol MJ, de Melker HE, Mollema L, van der Klis FRM (2018). Socioeconomic status is associated with antibody levels against vaccine preventable diseases in the Netherlands. Front Public Health.

[13] Zimmermann P, Curtis N (2019). Factors that influence the immune response to vaccination. Clin Microbiol Rev.

[14] Kennedy NA, Lin S, Goodhand JR, Chanchlani N, Hamilton B, Bewshea C (2021). Contributors to the CLARITY IBD study. Infliximab is associated with attenuated immunogenicity to BNT162b2 and ChAdOx1 nCoV-19 SARS-CoV-2 vaccines in patients with IBD. Gut.

[15] Hippisley-Cox J, Coupland CA, Mehta N, Keogh RH, Diaz-Ordaz K, Khunti K (2021). Risk prediction of COVID-19 related death and hospital admission in adults after COVID-19 vaccination: national prospective cohort study. BMJ.

[16] Tegally H, Wilkinson E, Giovanetti M, Iranzadeh A, Fonseca V, Giandhari J (2021). Detection of a SARS-CoV-2 variant of concern in South Africa. Nature.

[17] Gallo Marin B, Aghagoli G, Lavine K, Yang L, Siff EJ, Chiang SS (2021). Predictors of COVID-19 severity a literature review. Rev Med Virol.

[18] Wynants L, Van Calster B, Collins GS, Riley RD, Heinze G, Schuit E (2020). Prediction models for diagnosis and prognosis of covid-19: systematic review and critical appraisal. BMJ.
